# Turn-On Fluorescence Chemical Sensing through Transformation of Self-Trapped Exciton States at Room Temperature

**DOI:** 10.1021/acssensors.2c00964

**Published:** 2022-08-10

**Authors:** Yang Zhang, Samraj Mollick, Michele Tricarico, Jiahao Ye, Dylan Alexander Sherman, Jin-Chong Tan

**Affiliations:** Multifunctional Materials & Composites (MMC) Laboratory, Department of Engineering Science, University of Oxford, Parks Road, Oxford OX1 3PJ, U.K.

**Keywords:** turn-on fluorescent sensor, self-trapped exciton states, perylene, metal−organic framework, luminescent guest@MOF

## Abstract

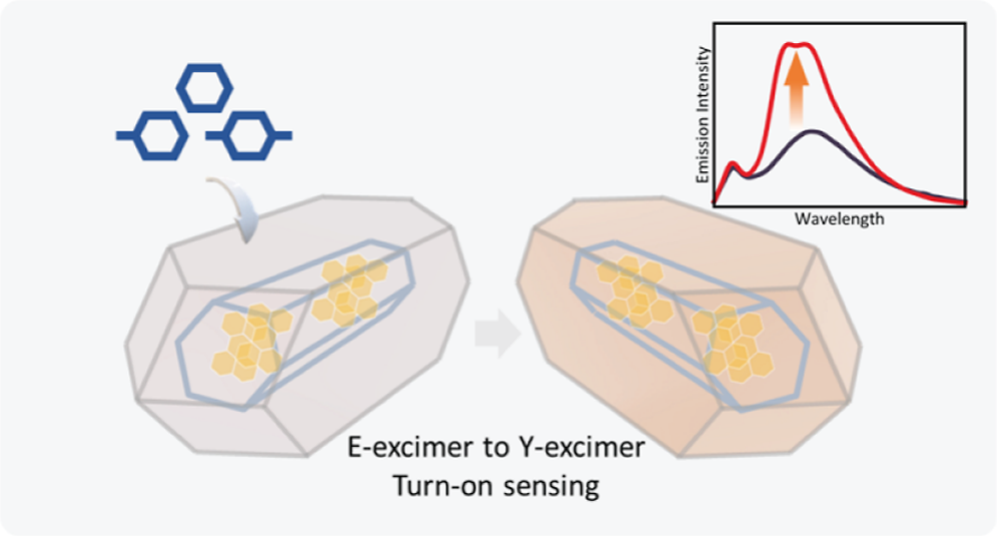

Most of the current fluorescence sensing materials belong to the turn-off type, which make it hard to detect toxic substances such as benzene, toluene, and xylene (BTX) due to the lack of active chemical sites, thereby limiting their development and practical use. Herein, we show a guest–host mechanism stemming from the confined emitter’s self-trapped exciton (STE) states or electron–phonon coupling to achieve turn-on fluorescence. We designed a luminescent guest@metal–organic framework (LG@MOF) composite material, termed perylene@MIL-68(In), and established its E-type excimeric emission properties in the solid state. Upon exposure to BTX, especially xylene, we show that the E-excimer readily converts into the Y-excimer due to nanoconfinement of the MOF structure. Such a transformation elevates the fluorescence intensity, thus realizing a turn-on type fluorescent sensor for detecting BTX solvents. Our results further demonstrate that controlling the STE states of perylene at room temperature (vs the previous report of <50 K) is possible *via* nanoscale confinement, paving the way to enabling turn-on type luminescent sensors for engineering practical applications.

Fluorescence sensing has recently become a popular research direction because of its simplicity, portability, rapid response, high selectivity, and high sensitivity.^1−4^ It works mainly through weakening of the fluorescence intensity (i.e., quenching mechanism) of the materials by affecting the photoinduced electron transfer,^5^ Förster resonance energy transfer,^6,7^ and/or charge transfer^8^ to achieve “turn-off” type fluorescence sensing.

Although the turn-off type sensing by fluorescence quenching has its own advantages,^9^ such as the ease of practical implementations,^2^ this approach poses two major limitations which are worth addressing. The first is that the side effects of environmental interference cannot be efficiently ruled out. For example, molecules in the environment, such as water moisture, may also cause a decrease in emission intensity.^10,11^ The second limitation is that it is hard to produce a fluorescent sensor to detect molecules that do not have active chemical sites,^12^ such as “BTX”, namely, benzene, toluene, and xylene. However, the capability for BTX sensing is urgently needed because exposure to these kinds of substances presents the risk of reproductive toxicity, and it is prevalent in both industrial sectors and in our daily life.^13−17^

To address the foregoing problems, herein, we demonstrate a novel “turn-on” type fluorescence sensing for the detection of BTX molecules without active chemical sites; we accomplished this by controlling the self-trapped exciton (STE) states of the emitters. Theoretically, the analyte may affect the coupling effect between excitons and phonons, thereby resulting in different fluorescent responses that can be harnessed for sensor applications.

In terms of the STE, perylene is a good starting point to discuss. This material is widely used in light-emitting diodes,^18,19^ photovoltaics,^20^ and organic field effect transistors.^21^ More importantly, perylene possesses two different STE states (Y- and E-states) with different fluorescence properties.^18,22−24^ Nevertheless, it is hard to use perylene itself as a sensor. The reasons are that perylene is susceptible to the aggregation-caused quenching (ACQ) effect; it thus has no fluorescence in the solid-state form, and the solubility of perylene is not high in many solvents, making it challenging to form a dimeric structure or STE states.^25^

Because of their porous, ordered, and highly adjustable crystalline structures, metal–organic frameworks (MOFs) are believed to be one of the most promising materials to combine with perylene to yield tunable luminescent sensing properties.^26−30^ In principle, the pores/channels of the MOF “host” can be used to encapsulate and isolate perylene “guest” molecules to overcome the ACQ effect, and when confined within an MOF structure, the solubility of perylene is no longer a concern.^31^ Of note, the emerging concept of the confinement of a luminescent guest (LG) in an MOF host, conferring an “LG@MOF” composite system, has huge potential for designing and engineering unconventional turn-on type luminescent sensors and lighting devices.^9^

In this work, we demonstrate a fluorescent sensing LG@MOF material by encapsulating perylene into the easy-to-synthesize and stable Materials of Institut Lavoisier-68(In) [MIL-68(In)]. For this perylene@MIL-68(In) composite, we show that perylene exhibits the E-state excimer fluorescence in the solid-state powder form, and it changes to the Y-state excimer emission when exposed to BTX, resulting in a prominent turn-on type sensing response. To the best of our knowledge, this kind of turn-on sensing from the transformation of STE states is the first example realized in the research field of LG@MOFs.^9^ Significantly, it is also the first time the transformation of the perylene E-state to the Y-state is evidenced under the room-temperature condition.

## Synthesis and Structure of the Perylene@MIL-68(In) Composite System

The perylene@MIL-68(In) system was synthesized by using the simple one-pot high-concentration reaction (HCR) method, a facile approach first described by Chaudhari et al.^32,33^ Full details of the synthetic steps for perylene@MIL-68(In) are given in the Experimental Section. It is worth mentioning that due to the deprotonation of triethylamine (TEA), this is the first method to produce MIL-68(In) at room temperature. The resulting composite materials were subsequently characterized by powder X-ray diffraction (PXRD), attenuated total reflection Fourier transform infrared spectroscopy (ATR–FTIR), synchrotron radiation infrared (SR-IR) spectroscopy, nanoindentation, and scanning electron microscopy (SEM).

**Figure 1:**
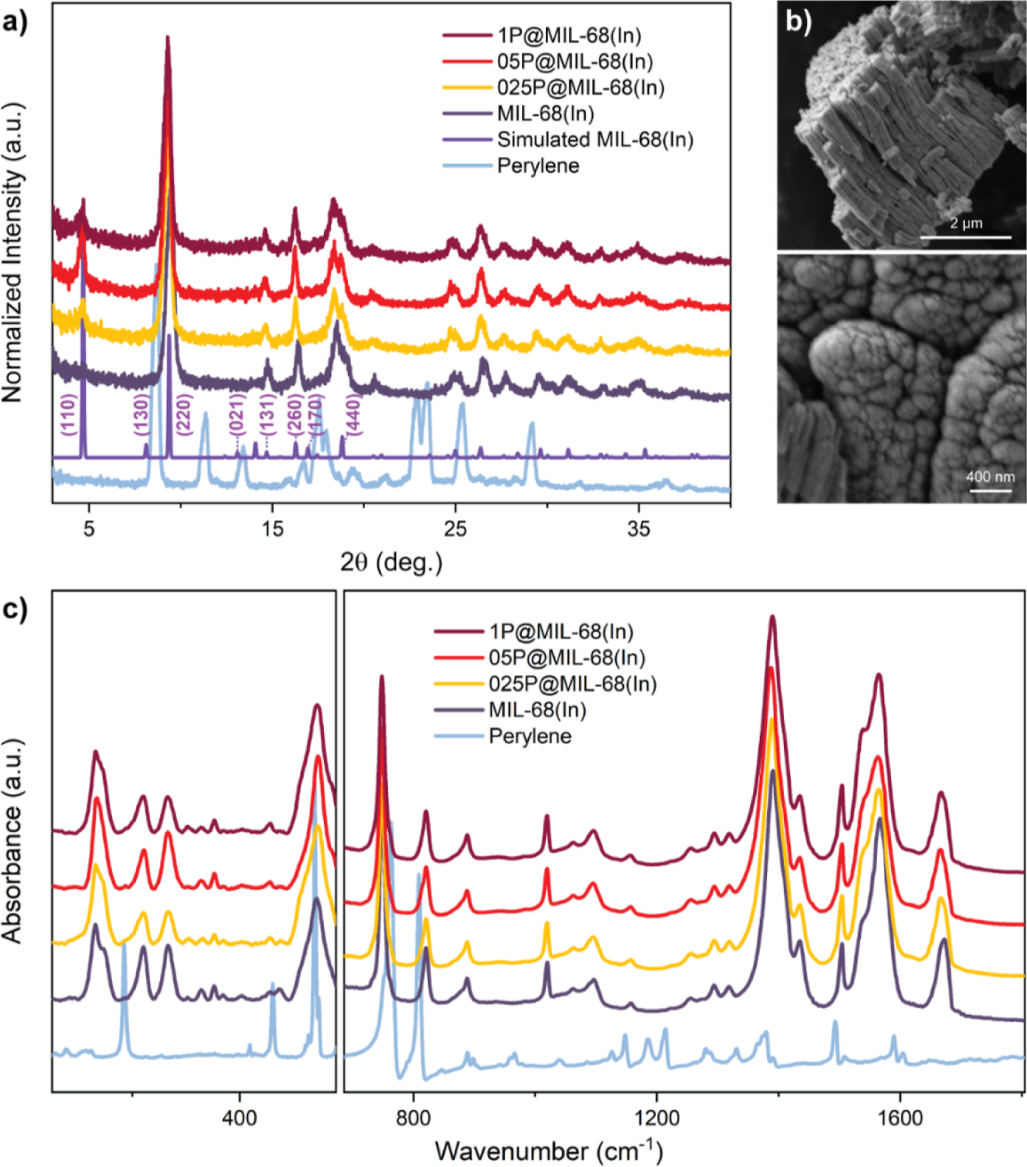
(a) Comparison of the XRD patterns of the simulated vs synthesized MIL-68(In) and perylene@MIL-68(In) featuring three perylene concentrations (1P, 05P, and 025P show that the amount of perylene used in the synthesis is 1, 0.5, and 0.25 mmol, respectively). (b) Field-emission SEM (FESEM) images of MIL-68(In), where the upper panel shows the rod-like nanocrystals in the axial direction, while the lower panel shows the morphology in the transverse direction. (c) FTIR results of MIL-68(In) and perylene@MIL-68(In) (left: synchrotron-radiation-FTIR; right: ATR–FTIR).

From Figure 1a, it can be seen that the PXRD patterns of the prepared MIL-68(In) MOF host and perylene@MIL-68(In) are consistent with the simulations, which indicate that MIL-68(In)’s crystal structure is successfully generated by the HCR method, and the introduction of perylene molecules does not hinder the crystal formation of the MOF host.^34^ The same conclusion can also be drawn from the FTIR results (Figure 1c) due to the high similarity between the spectra of MIL-68(In) and perylene@MIL-68(In). The nanoindentation results show that the hardness of the crystal increased by ∼20% after the encapsulation of perylene (Figure S1 and Table S1), suggesting that the formation of perylene@MIL-68(In) may have given rise to an interstitial hardening effect. SEM images (Figures 1b and S2) show that the synthesized MIL-68(In) and perylene@MIL-68(In) possess nanosized columnar crystals, which coincide with the typical crystal morphology of MIL-68 produced with a long reaction time (hours) at a high temperature (>55 °C).^35^ Together, our results demonstrate that the HCR method can produce MIL-68(In) immediately under a significantly milder reaction condition, which is potentially useful for future commercialization.

## Luminescent Properties of Perylene@MIL-68(In)

**Figure 2:**
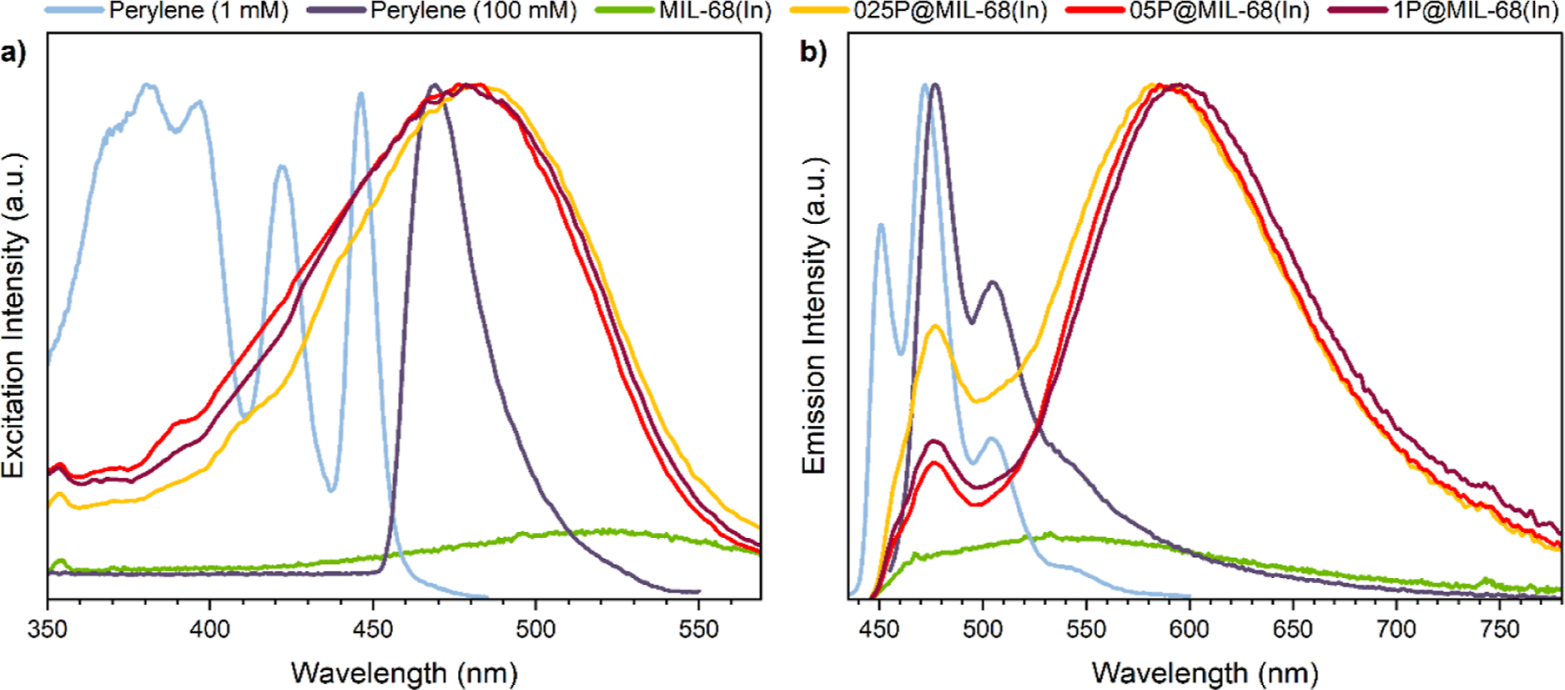
Normalized (a) excitation spectra (measured under em@600 nm) and (b) emission spectra (measured under ex@440 nm) of perylene solutions, MIL-68(In) powders, and perylene@MIL-68(In) in the solid state. Note: MIL-68(In) is not normalized due to its weak signal.

We then performed photophysical characterization of perylene@MIL-68(In) with different amounts of perylene guest loading. The excitation and emission spectra are shown in Figure 2a,b, respectively. For control, a physical mixture of perylene + MIL-68(In) was also prepared; notably, this sample exhibits emission (Figure S3) completely different from the perylene@MIL-68(In) samples obtained by the HCR encapsulation method. It is apparent that the fluorescence properties of the perylene@MIL-68(In) systems are derived from perylene molecules because pure MIL-68(In) is virtually nonemissive at the selected excitation wavelength of 440 nm. For perylene@MIL-68(In) at all three concentrations, their emission peaks can be divided into two parts: (1) a small peak at around 475 nm and (2) a broad and intense peak at around 600 nm. On the basis of previous studies on perylene,^18,22−25^ we reasoned that the first peak originates from the emission of free excitons, and the second peak is attributed to the STE emission. These photophysical characteristics indicate that the α-perylene-like structure is generated inside the MIL-68(In) channel and emits as the E-state excimer.^36^ Furthermore, the lifetime data of the system obtained by using the time-correlated single photon counting (TCSPC) technique (Table S2 and Figure S4) show a lifetime component (τ_4_) that is around 18 ns, further supporting the existence of the E-state excimer. It is worth mentioning that the fluorescence performance of perylene solutions with different concentrations verified our theoretical hypothesis in the introduction part: perylene is challenging to form a dimeric structure and induce excimer emission due to the solubility problem, but MOFs can help to overcome this limitation through confinement in nanoscale pores/channels.

**Scheme 1:**
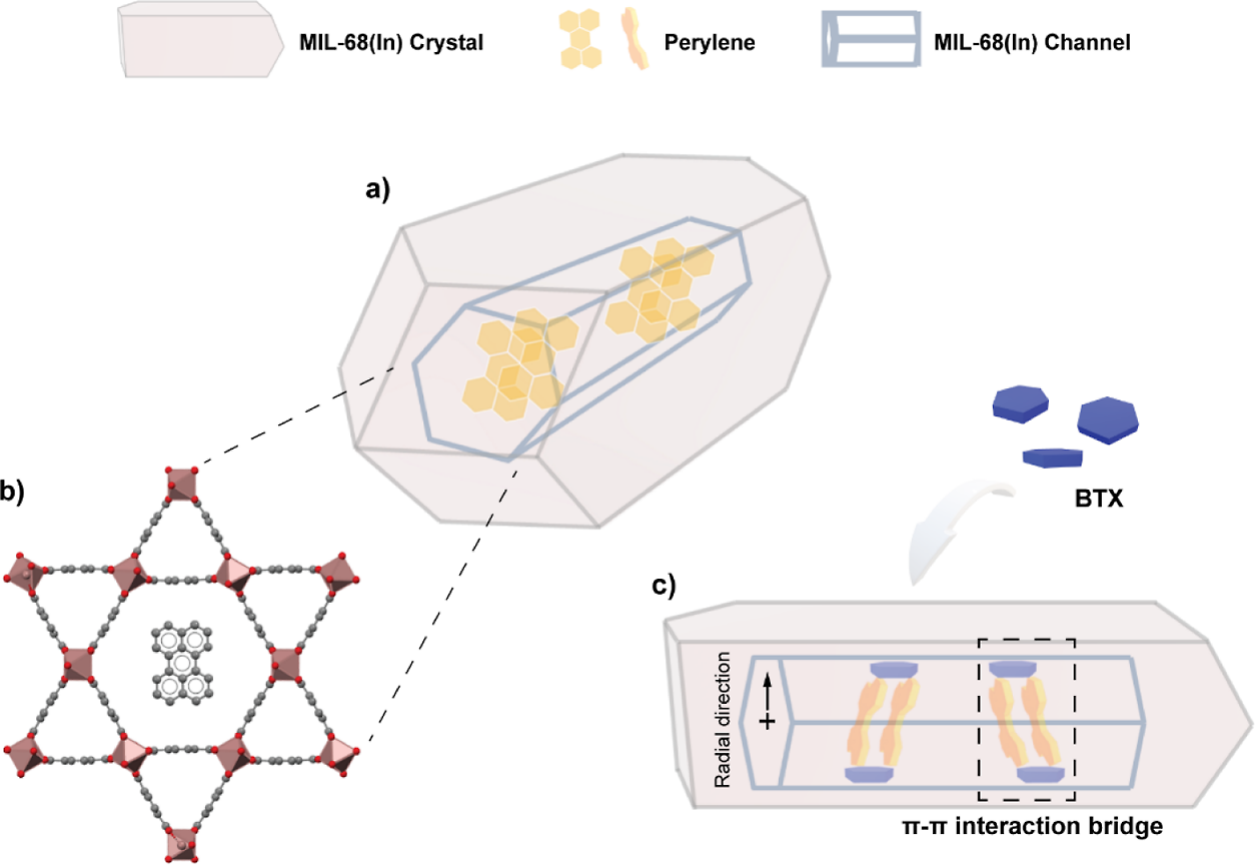
(a,b) LG@MOF Crystal Structure of Perylene@MIL-68(In) and Its Initial Molecular Configuration under Confinement of One-Dimensional Channels and (c) Proposed Sensing Mechanism of Perylene@MIL-68(In) Subject to BTX Molecules. Color Scheme: Indium in Dark Red, Carbon in Gray, and Oxygen in Red

Considering the E-state emission characteristic, the channel size of MIL-68(In) (16 and 6 Å), and the previous research outcome in ref (22), it is reasonable to infer that in the channel of MIL-68(In), perylene molecules exist in a disordered dimeric structure. This means that the longest axis of perylene molecules tilts in the radial direction of MIL-68(In) channels, as shown in Scheme 1. This kind of structural alignment of confined guests also explains the incremental red shift evidenced for the 025P, 05P, and 1P@MIL-68(In) samples (Figure 2b) because the dimeric perylene can interact with the adjacent perylenes, and the interaction will be enhanced with an increase of perylene amount trapped within the MOF channel. The decrease in quantum yields (QYs, Table S3) and excimer lifetime (τ_4_, Table S2 and Figure S4) when the perylene amount increases also supports the proposed structure as the enhanced interactions will increase the non-radiative decay.^26,30^

## BTX Sensing Performance of Perylene@MIL-68(In)

**Figure 3:**
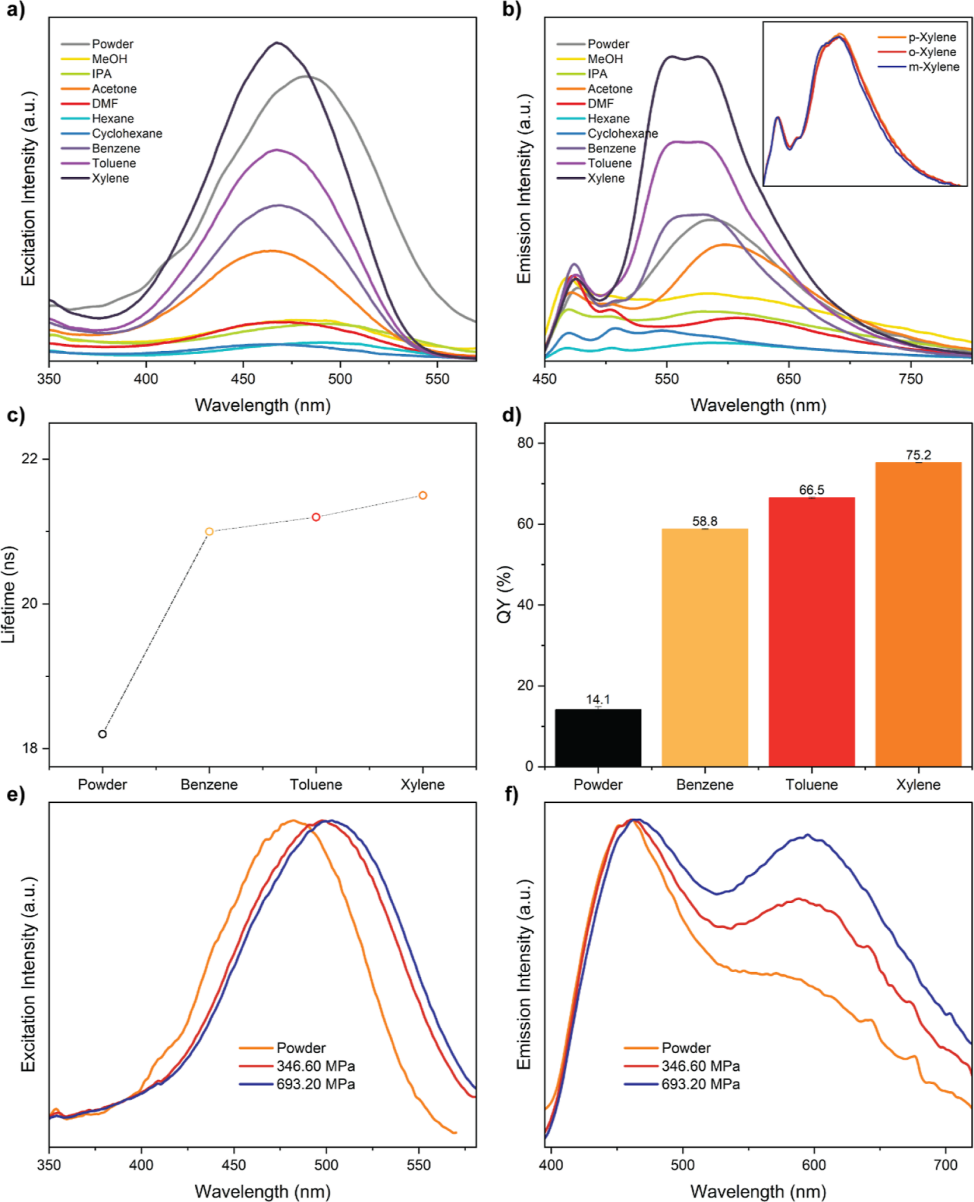
(a) Excitation (measured under em@600 nm) and (b) emission spectra (measured under ex@440 nm) of 025P@MIL-68(In) in different solvents. The inset of (b) shows the emission spectra of 025P@MIL-68(In) in *p*-, *o*-, and *m*-xylene. (c) Lifetimes (excimer component) and (d) QYs of 025P@MIL-68(In) powders and when subject to BTX (the QY of each sample was tested three times to determine the average and standard deviations). (e) Normalized excitation (measured under em@600 nm) and (f) emission spectra (measured under ex@380 nm) of 025P@MIL-68(In) and its pellets subjected to two different nominal pressures.

Subsequently, we tested the BTX sensing properties of the perylene@MIL-68(In) system. In order to better observe the subtle changes, we chose 025P@MIL-68(In) with the least amount of perylene and the highest QY for testing. As shown in Figure 3, perylene@MIL-68(In) delivers a prominent turn-on sensing response with a slight blue shift and splitting of the emission peak (∼600 nm) when exposed to the BTX molecules in solution. On the contrary, when exposed to DMF/acetone, polar aprotic electron-deficient solvents, the peak at 600 nm exhibits a red shift with a decline in its intensity.

The rising intensity and splitting of the peak at ∼600 nm (Figure 3b) can be explained by the perylene in the MIL-68(In) channels undergoing a transformation from the E-state to Y-state.^24^ This kind of transformation can be attributed to the formation of the π–π interaction bridge shown in Scheme 1. When the perylene inside encounters BTX, electron-rich solvents, the organic linker of MIL-68(In) benzenedicarboxylate (BDC), BTX, and dimeric perylene form a relatively strong π–π interaction “chain”, thereby inducing an effect similar to the lattice confinement effect.^24,26,37,38^ In this situation, the electron–phonon coupling of the E-state will be affected to generate the Y-state. Compared with the single emission characteristic of the E-state, Y-emission itself possesses multiple luminescent peaks, a higher peak intensity, and a shorter peak wavelength.^24^ Therefore, when exposed to BTX, the perylene@MIL-68(In) peak showed an intensity enhancement, splitting, and blue shift. The deconvolution result of the emission peak (Figure S5) reveals the typical feature of the Y-excimer, in which the fitted peaks denoted as 2, 3, and 4 may correspond to the emission of the Y-excimer reported in ref (24).

Furthermore, based on this, the occurrence of a higher peak intensity and more pronounced peak splitting upon contact with xylene (compared to that with benzene and toluene) can also be understood. As the electron-donating ability of BTX becomes stronger (i.e., benzene < toluene < xylene), the π–π interaction bridge will become stronger correspondingly, resulting in a more substantial confinement effect for perylene in the channel.^39^ In addition, xylene was then selected to test the sensitivity, selectivity, and reversibility of the system (see Figure S6, including the limit of detection value). It is worth mentioning that the Y-emission can only be observed at temperatures below 50 K for pure perylene.^24^ Remarkably, herein, we show for the first time that, thanks to the LG@MOF assembly, the transformation between the E-state and Y-state is realized at room temperature.

To further validate the proposed theory, we performed the lifetime and QY measurements on perylene@MIL-68(In) (Figure 3c,d). The lifetime data shown in Figure 3c, Table S4, and Figure S7 suggest that the 18 ns time component (belonging to the E-state) increases when contacting BTX solvents.^25^ Meanwhile, the QY also shows an increasing trend. More importantly, this rise can be associated with BTX’s electron-donating ability (Figure 3b–d): the higher the electron-donating ability, the larger the lifetime and QY. These phenomena indicate that the non-radiative decay of perylene in MIL-68(In) is reduced due to the π–π interaction bridge, thus inducing a strong confinement effect and proving the transformation from the E- to Y-emissions.

The fluorescence performance of perylene@MIL-68(In) subject to different compressive pressures also supports our theory. According to the previous studies,^26,38,39^ it can be deduced that when the pressure increases, the π–π interaction between the linker (BDC) and perylene will increase, thus causing perylene to become more tightly constrained. The excitation and emission spectra of perylene@MIL-68(In) under pressure (Figure 3e,f) illustrate that when the interaction is enlarged, the peak intensity corresponding to the perylene excimer increases. Furthermore, the τ_4_ excimer lifetime also increases sharply when pressure rises (Table S5 and Figure S8), compared with that of the τ_1_ to τ_3_ lifetime components which are relatively unchanged. Therefore, it is shown that enhancing the π–π interaction in this LG@MOF system does enhance the confinement effect and hence results in a stronger excimer emission. The pressure-dependent luminescence data further substantiate the rational of our proposed theory from another standpoint.

By way of comparison, the behavior of perylene@MIL-68(In) when exposed to electron-deficient solvents can be explained by the proposed theory as well. When the system is in DMF/acetone solvents, the emission at ∼600 nm shows a relative decline and red shift (Figure 3b). In Table S4 and Figure S7, the perylene@MIL-68(In) lifetime is smaller in DMF and acetone than in BTX. Based on the theory, it can be interpreted that electron-deficient solvents reduce the π–π interaction between the BDC linkers and perylene, causing the decline in intensity and redshift of the emission peak.

## Conclusions

In conclusion, we have shown the facile synthesis of perylene@MIL-68(In) under ambient conditions by harnessing the HCR method. Of note, perylene@MIL-68(In) exhibits the E-excimer emission characteristics in the solid state. When exposed to BTX, the perylene molecules present in the MIL-68(In) channels will receive a strong confinement effect and affect the STE states. This guest–host confinement effect switches the E-state to Y-state emission, resulting in the turn-on fluorescent response when subject to the electron-rich BTX solvents. The sensing mechanism proposed here using the transformation between different STE states is the first exemplar in the field of LG@MOF research.^9^ The simple synthesis method and the uncommon turn-ontype sensing behavior have opened up a new approach for developing highly selective fluorescent sensors.

## Experimental Section

### Synthesis of Perylene@MIL-68(In) and MIL-68(In) by Using the HCR Method

The synthesis was accomplished by leveraging the HCR method.^32,33^ 15 mL of a dimethylformamide (DMF) solution of 4.8 mmol BDC and TEA (9.6 mmol) was combined with 50 mL of a dichloromethane (DCM) solution of 0.25/0.5/1 mmol perylene. After the combination, 15 mL of a DMF solution of 4.8 mmol indium nitrate was immediately added into the mixture. Then, the product was formed instantly and washed thoroughly five times (two times with DCM, two times with DMF, and one time with methanol) to remove the excess guests adhered to the external MOF surfaces. The nanocrystals of perylene@MIL-68(In) were separated from the suspension by centrifugation at 8000 rpm for 10 min.

### Sample Preparation for Fluorescence Characterization

The pellets for the mechanofluorochromic study were made using a manual hydraulic press (Specac Atlas) with a 1.2 cm diameter die under a uniaxial compressive force of 4 and 8 tones. Perylene@MIL-68(In) suspensions for the solvatochromic study were prepared by diluting 5 mg of perylene@MIL-68(In) in 8 mL of the solvent. For sensitivity and selectivity research, 5 mg of perylene@MIL-68(In) was first dispersed in 8 mL of cyclohexane, and then, 2 mL of the solution was used as the base solution for the subsequent measurements.

### Materials Characterization

The crystal morphologies and structures were examined using a field-emission scanning electron microscope (FESEM LYRA_3_ GM TESCAN). PXRD patterns were recorded using a Rigaku MiniFlex with a Cu Kα source (1.541 Å). The nanoindentation tests were conducted using an iMicro nanoindenter (KLA-Tencor). Steady-state fluorescence spectra, lifetimes, and QYs were recorded using the FS-5 spectrofluorometer (Edinburgh Instruments). For TCSPC lifetime measurement, a 445 nm laser was used; the stop condition was set to be at 10,000 counts, and a filter was used to avoid the interference of scattering light. ATR–FTIR spectra were recorded using a Nicolet iS10 FTIR spectrometer.

### SR-IR Spectroscopy

High-resolution SR-IR vibrational spectra of all compounds were recorded at the multimode IR imaging and microspectroscopy (MIRIAM) Beamline B22 at the Diamond Light Source synchrotron. Measurements were performed in vacuum *via* a Bruker Vertex 80v FTIR spectrometer with an ATR accessory (Bruker Optics, Germany). For the far-IR spectral range below 700 cm^–1^, a bolometer cooled by liquid helium was used for the detection of terahertz signals. All spectra were acquired with a resolution of 4 cm^–1^ and a scanner velocity of 20 kHz.

## Supporting Information Available

Nanoindentation results; SEM images; emission properties of the physically mixed sample; lifetime decay curves and lifetime data; QYs; and sensitivity, selectivity, and reversibility data (PDF)
