# Supplementary material for: Turn-On Fluorescence Chemical Sensing through Transformation of Self-Trapped Exciton States at Room Temperature
Source: ACS Sens. 2022 Aug 10;7(8):2338–44. doi: 10.1021/acssensors.2c00964 (PMC9425555; doi:10.1021/acssensors.2c00964)
Supplement: Supplementary file 1 — se2c00964_si_001.pdf [file se2c00964_si_001.pdf]

*Supporting Information*

*for*

# Turn-on fluorescence chemical sensing through transformation of self-trapped exciton states at room temperature

*Yang Zhang, Samraj Mollick, Michele Tricarico, Jiahao Ye, Dylan Alexander Sherman, and Jin-Chong Tan**

Multifunctional Materials & Composites (MMC) Laboratory, Department of Engineering Science, University of Oxford, Parks Road, Oxford OX1 3PJ, United Kingdom.

*Corresponding author
E-mail: jin-chong.tan@eng.ox.ac.uk

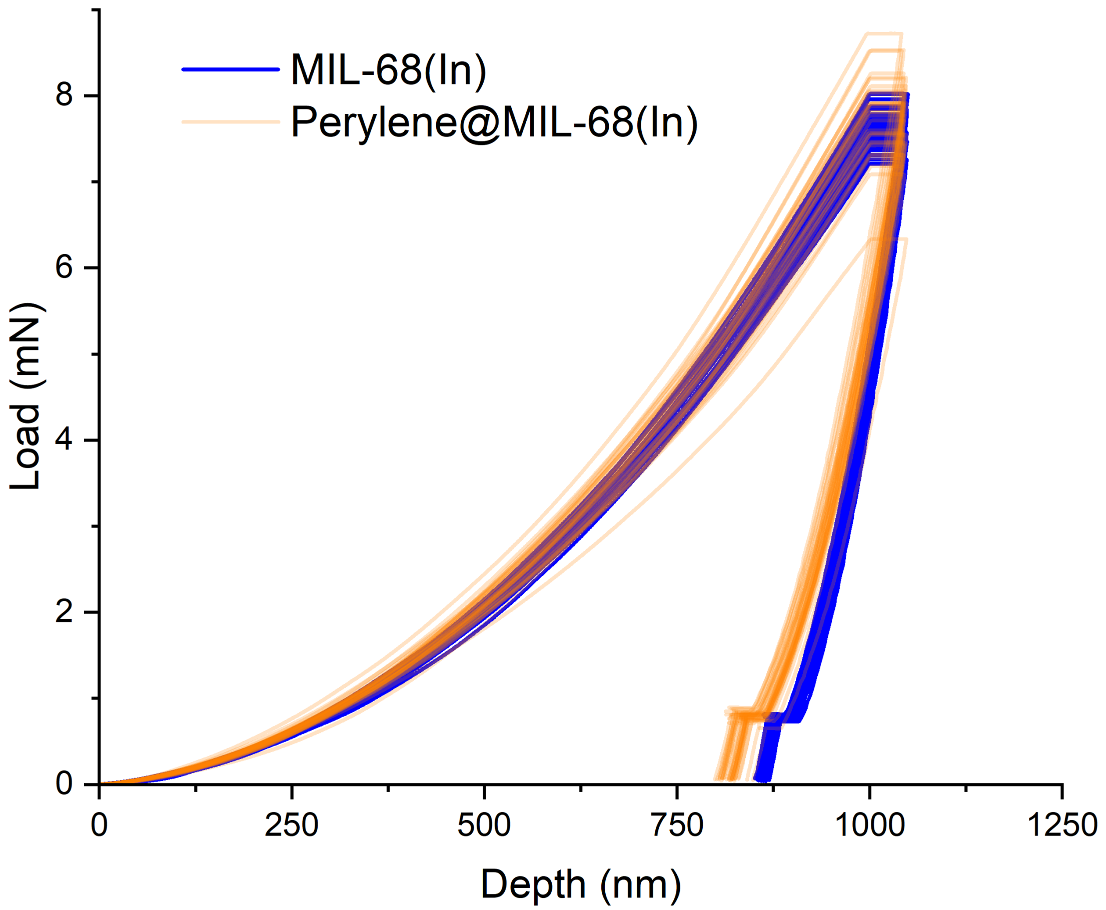


**Figure S1.** Load-depth curves resulting from nanoindentation tests of MIL-68(In) and perylene@MIL-68(In) with a Berkovich indenter. The results are summarized in Table S1.

**Table S1.** Indentation modulus and hardness measurements (CSM method) obtained from Berkovich nanoindentation. Mean values and standard deviations from this work were calculated from an indentation depth range of 200 – 1000 nm.

| Sample | # of indents | Indentation modulus*, *M* (GPa) | Hardness, *H* (MPa) |
|---|---|---|---|
| MIL-68(In) | 32 | 13.24±0.52 | 402±13 |
| Perylene@MIL-68(In) | 32 | 11.74±0.66 | 486±32 |

* Let Poisson's ratio, $\nu = 0$.

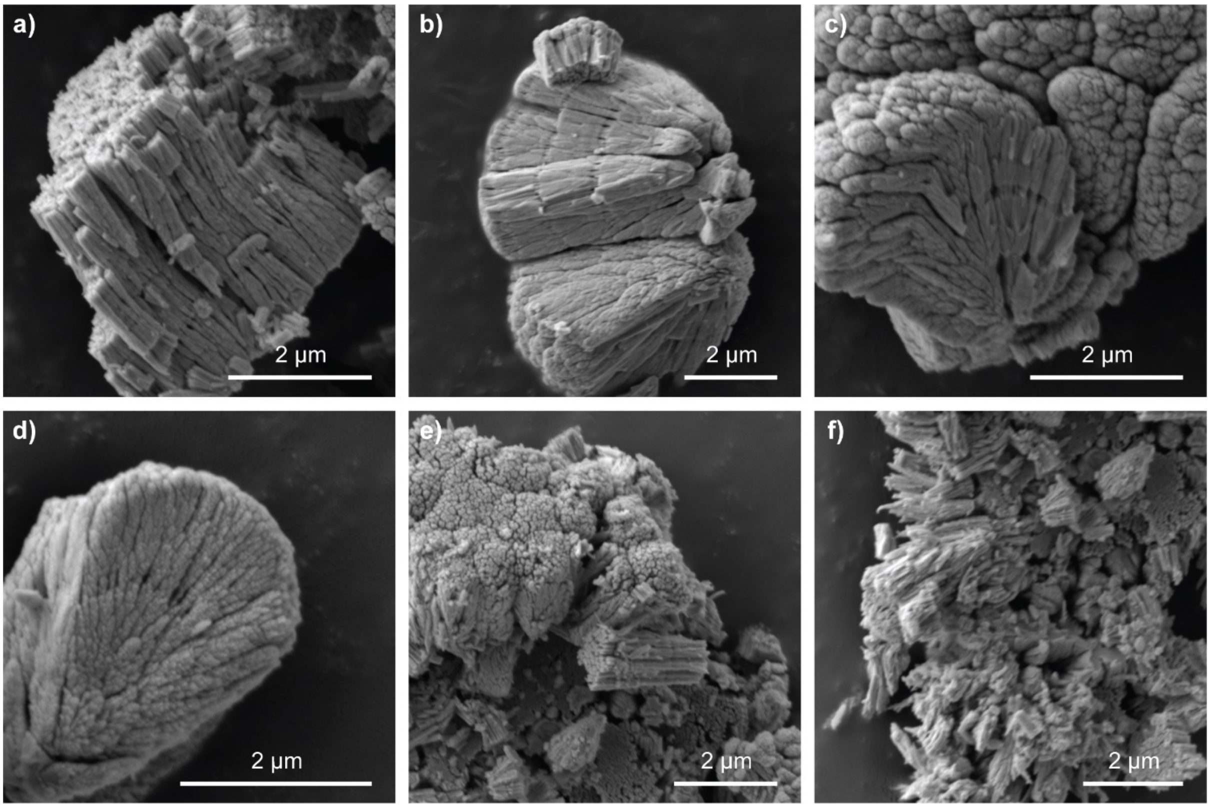


**Figure S2.** FESEM images of (a-c) MIL-68(In), (d) 025P@MIL-68(In), (e) 05P@MIL-68(In), and (f) 1P@MIL-68(In). 1P, 05P, and 025P designate the amount of perylene used in the synthesis to be 1 mmol, 0.5 mmol, and 0.25 mmol, respectively.

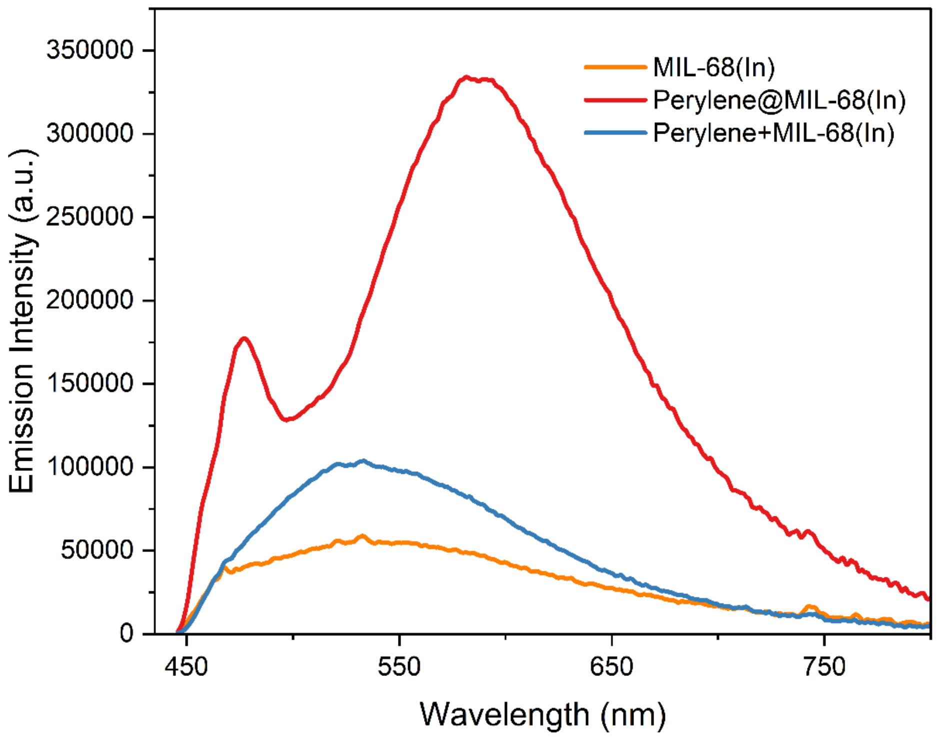


**Figure S3.** Comparing the emission properties of MIL-68(In), perylene@MIL-68(In), and physically mixed perylene+MIL-68(In) [*i.e.* perylene guests predominantly adhered on the surface of MIL-68(In)]. The perylene+MIL-68(In) was made by simple blending (physical mixing) of the two constituents [in mortar and pestle by maintaining the same guest concentration as 025P@MIL-68(In)]. It is clear that the emission of perylene+MIL-68(In) is different from the perylene@MIL-68(In), which indicates that the perylene guests are mainly confined within the MOF channels in the perylene@MIL-68(In) system.

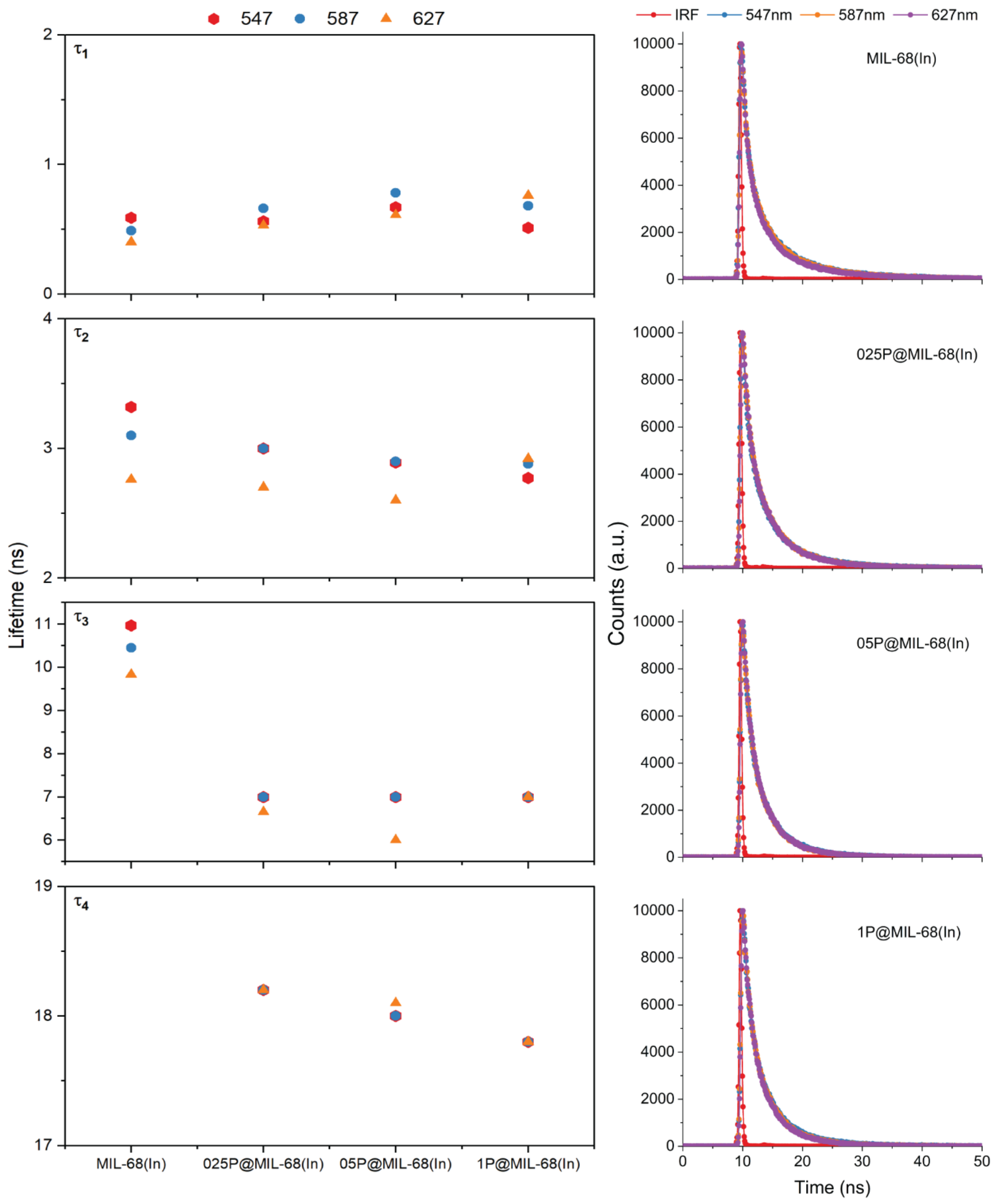


**Figure S4.** Lifetime data of MIL-68(In) and perylene@MIL-68(In) powders observed under the 547, 587, and 627 nm emission wavelength. IRF = instrument response function.

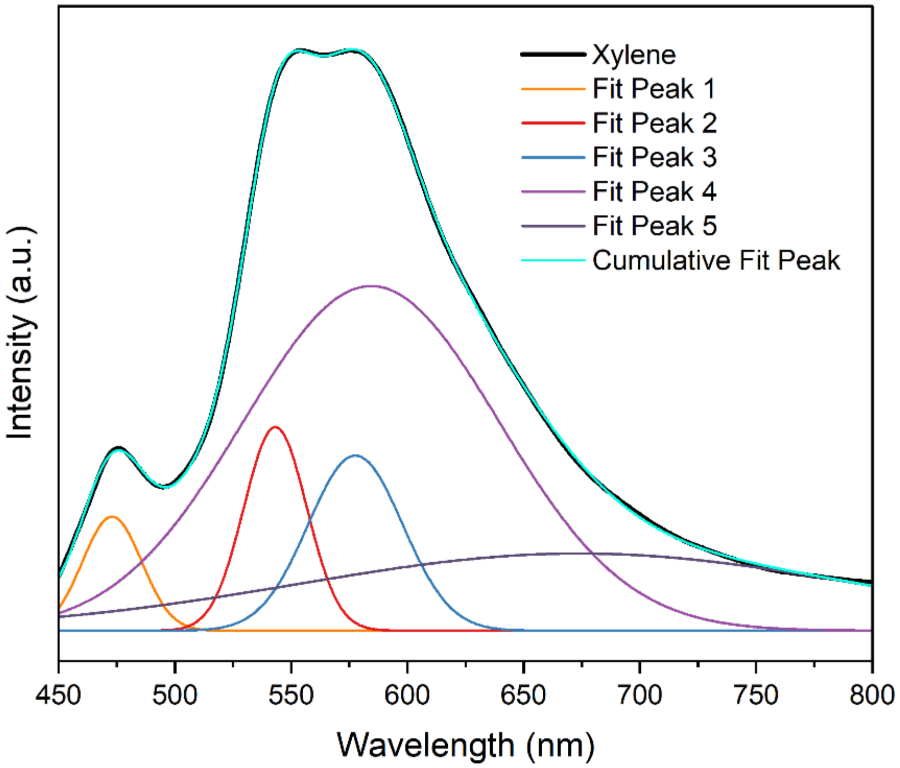


| Model | Gaussian | | | | |
|---|---|---|---|---|---|
| Equation | y = y0 + A/(w*sqrt(pi/(4*ln(2)))) * exp(-4*ln(2)*(x-xc)^2/w^2) | | | | |
| Plot | Peak1 (Intensity) | Peak2 (Intensity) | Peak3 (Intensity) | Peak4 (Intensity) | Peak5 (Intensity) |
| y0 | -26932.8 ± 33059.8 | -26932.8 ± 33059.8 | -26932.8 ± 33059.8 | -26932.8 ± 33059.8 | -26932.8 ± 33059.8 |
| xc | 473. 0 ± 0.1 | 543.2 ± 0.1 | 577.6 ± 0.3 | 584.4 ± 2.7 | 674.0 ± 58.2 |
| A | 2777278.4 ± 167134.3 | 5263344.9 ± 182305.5 | 6808557.9 ± 285976.7 | 36278100.0 ± 6350215.6 | 17824000.0 ± 25039400.0 |
| w | 30.0 ± 0.6 | 31.8 ± 0.3 | 47.9 ± 0.7 | 129.5 ± 4.9 | 284.2 ± 187.2 |
| Reduced Chi-Sqr | 1551025.018 | | | | |
| R-Square (COD) | 0.99992 | | | | |
| Adj. R-Square | 0.99992 | | | | |

**Figure S5.** Deconvolution of the emission spectrum of perylene@MIL-68(In) in xylene by using the Gaussian method implemented in OriginPro.

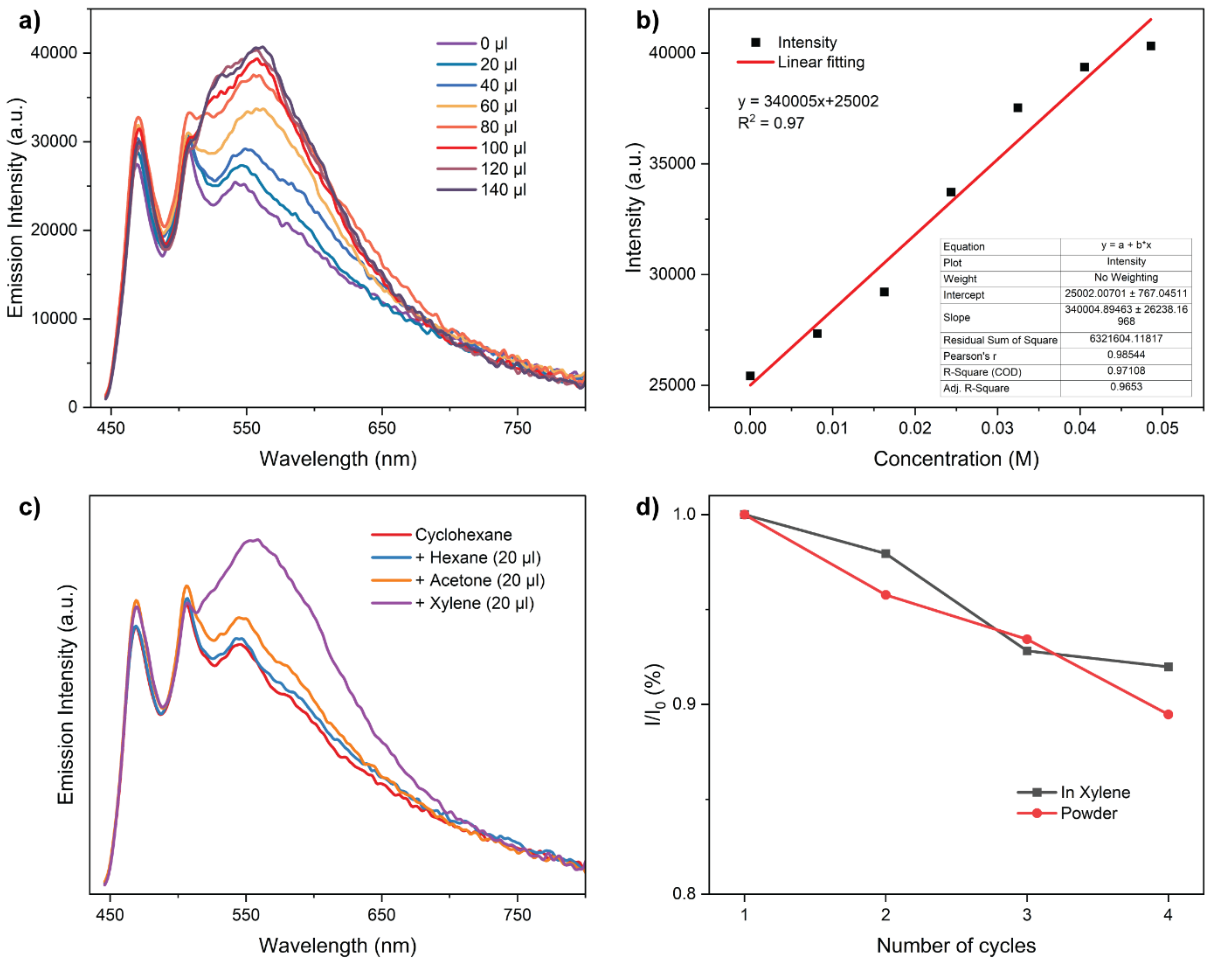


**Figure S6.** (a) Emission spectra and (b) peak intensities (~ 550 nm) of perylene@MIL-68(In) in cyclohexane in the presence of different concentrations of xylene. (c) Emission spectra of perylene@MIL-68(In) dispersed in cyclohexane and after the addition of different solvent analytes. (d) Reversibility of xylene sensing behavior.

The calculation of the limit of detection (LOD) based on the definition by IUPAC criterion [1]:

$$\text{Detection limit} = 3\sigma / \text{slope}$$

The slope can be obtained from Figure S6(b), and the standard deviation (σ) for the blank probe with the addition of xylene was calculated to be 268.69 (5 repeated measurements). Therefore, the LOD = 3*268.69/340005 = 0.002 M = 2 mM.

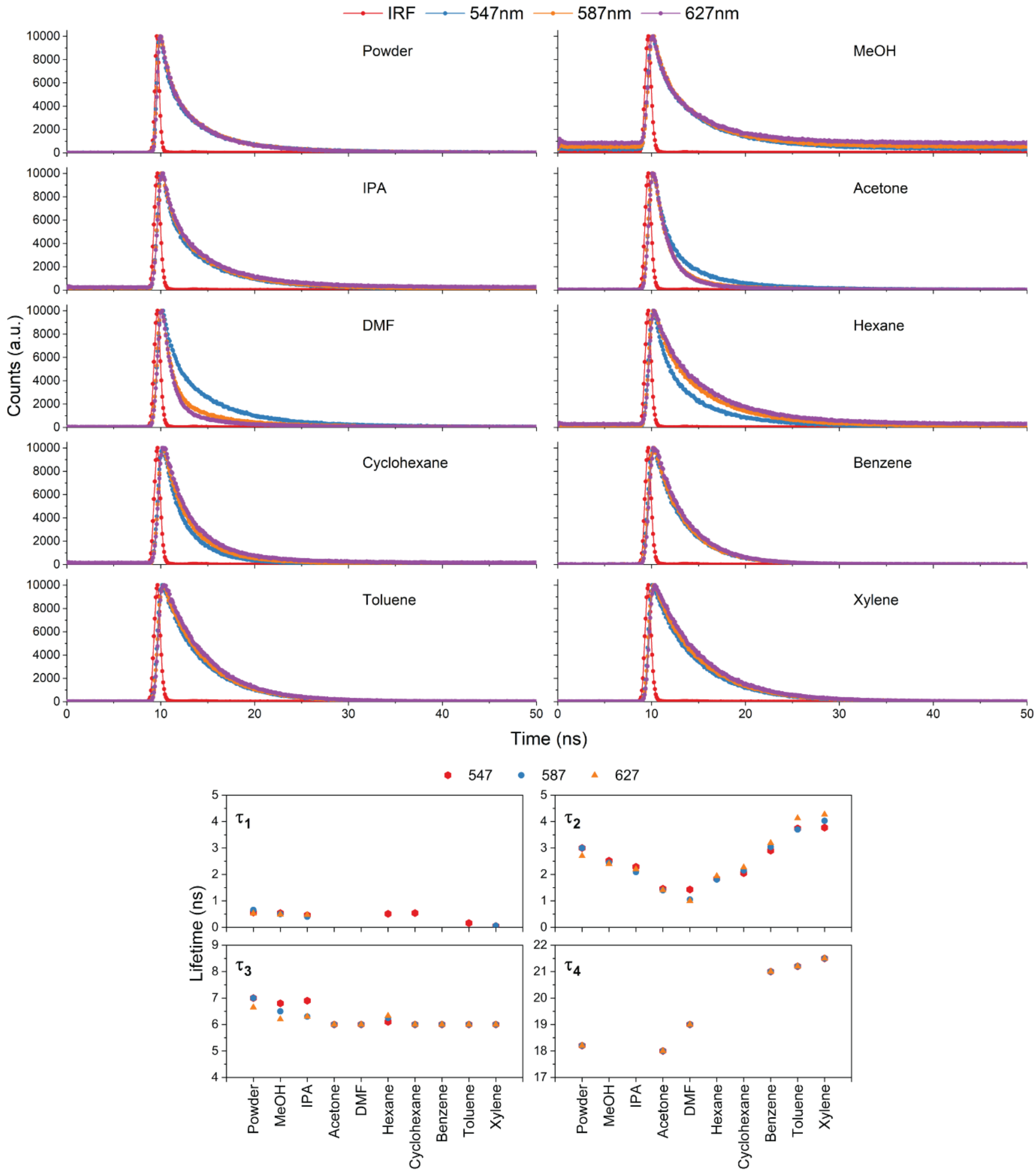


**Figure S7.** Lifetime data of perylene@MIL-68(In) in different solvents observed under the 547, 587, and 627 nm emission wavelength. IRF = instrument response function.

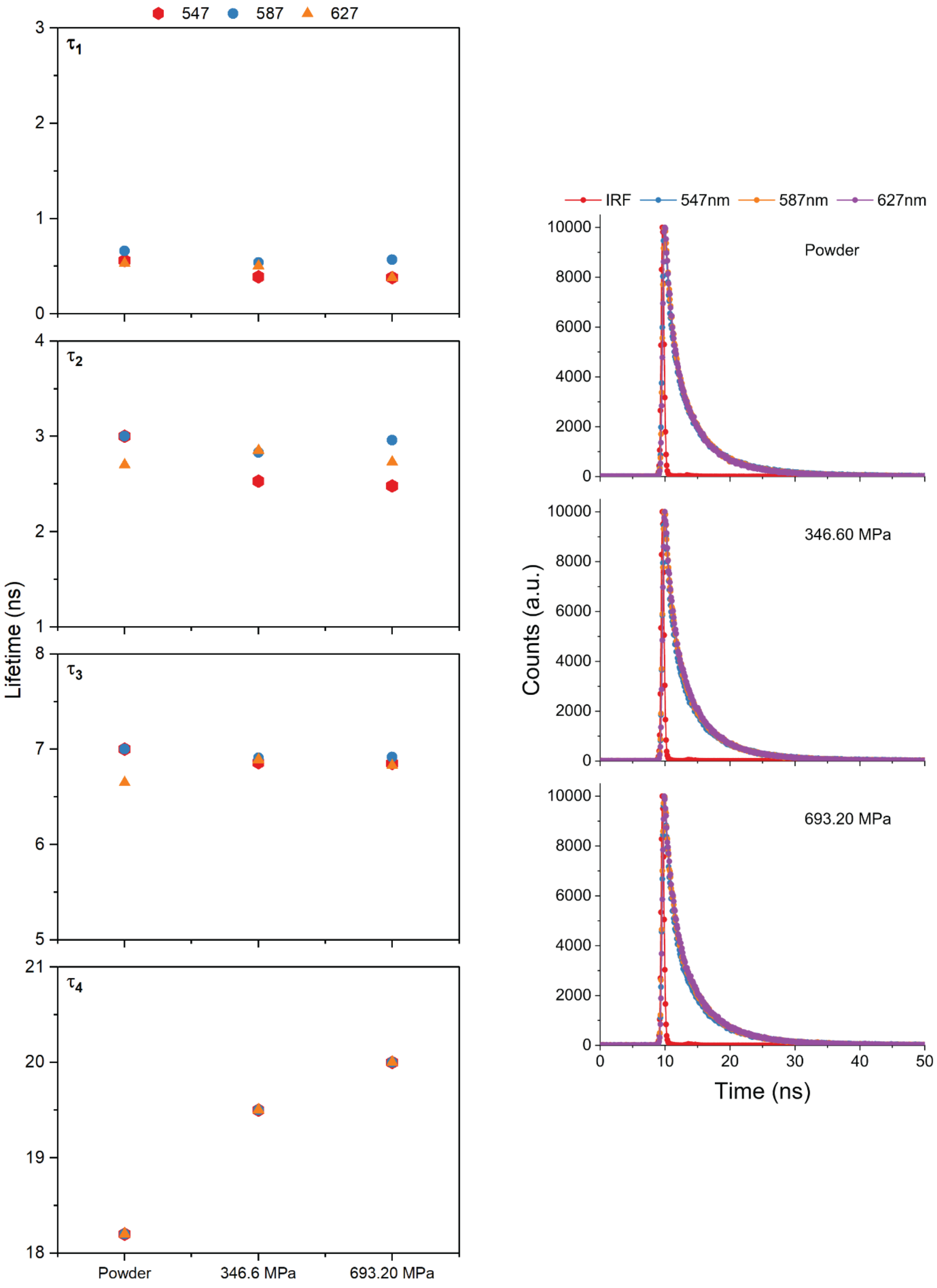


**Figure S8.** Lifetime data of perylene@MIL-68(In) pellets observed under the 547, 587, and 627 nm emission wavelength. IRF = instrument response function.

**Table S2.** Values of time constants ($\tau_i$) of the emission decay of MIL-68(In), 025P@MIL-68(In), 05P@MIL-68(In), and 1P@MIL-68(In) powder upon excitation at 445 nm. Since $\tau_1$, $\tau_2$, and $\tau_3$ of the perylene@MIL-68(In) systems are resembling those of MIL-68(In), it was not possible to distinguish the contribution of perylene from $\tau_1$, $\tau_2$, and $\tau_3$. However, $\tau_4$ belongs only to the perylene excimers. $R_t = \Sigma a_i e^{(-t/\tau_i)}$, $R_t$ is the quantity/counts at time *t*. The goodness-of-fit is given by $\chi^2$.

| **Sample** | **Wavelength (nm)** | **$\tau_1$ (ns)** | **$\tau_2$ (ns)** | **$\tau_3$ (ns)** | **$\tau_4$ (ns)** | **$\chi^2$** |
|---|---|---|---|---|---|---|
| MIL-68(In) | 547 | 0.59 | 3.32 | 10.97 | - | 1.350 |
| | 587 | 0.49 | 3.10 | 10.45 | - | 1.431 |
| | 627 | 0.40 | 2.76 | 9.83 | - | 1.324 |
| 025P@MIL-68(In) | 547 | 0.56 | 3.00 | 7.00 | 18.20 | 1.276 |
| | 587 | 0.66 | 3.00 | 7.00 | 18.20 | 1.200 |
| | 627 | 0.53 | 2.70 | 6.65 | 18.20 | 1.332 |
| 05P@MIL-68(In) | 547 | 0.67 | 2.89 | 7.00 | 18.00 | 1.270 |
| | 587 | 0.78 | 2.90 | 7.00 | 18.00 | 1.205 |
| | 627 | 0.61 | 2.60 | 6.00 | 18.10 | 1.301 |
| 1P@MIL-68(In) | 547 | 0.51 | 2.77 | 7.00 | 17.80 | 1.172 |
| | 587 | 0.68 | 2.88 | 7.00 | 17.80 | 1.354 |
| | 627 | 0.76 | 2.92 | 7.00 | 17.80 | 1.314 |

**Table S3**. Quantum yield (QY) of 1P@MIL-68(In), 05P@MIL-68(In), and 025P@MIL-68(In). The QY of each sample was tested three times to determine the average and standard deviation values.

| **Sample** | **QY (%)** |
|---|---|
| 1P@MIL-68(In) | $4.63 \pm 0.07$ |
| 05P@MIL-68(In) | $5.14 \pm 0.60$ |
| 025P@MIL-68(In) | $14.13 \pm 0.74$ |

**Table S4.** Values of time constants ($\tau_i$) of the emission decay of 025P@MIL-68(In) powder and under different solvents upon excitation at 445 nm.

| Sample | Wavelength (nm) | $\tau_1$ (ns) | $\tau_2$ (ns) | $\tau_3$ (ns) | $\tau_4$ (ns) | $\chi^2$ |
|---|---|---|---|---|---|---|
| Powder | 547 | 0.56 | 3.00 | 7.00 | 18.20 | 1.276 |
| | 587 | 0.66 | 3.00 | 7.00 | 18.20 | 1.200 |
| | 627 | 0.53 | 2.70 | 6.65 | 18.20 | 1.332 |
| MeOH | 547 | 0.54 | 2.52 | 6.80 | - | 1.386 |
| | 587 | 0.50 | 2.44 | 6.50 | - | 1.479 |
| | 627 | 0.49 | 2.40 | 6.20 | - | 1.461 |
| IPA | 547 | 0.46 | 2.29 | 6.90 | - | 1.442 |
| | 587 | 0.40 | 2.09 | 6.30 | - | 1.300 |
| | 627 | 0.47 | 2.20 | 6.30 | - | 1.175 |
| Acetone | 547 | - | 1.46 | 6.00 | 18.00 | 1.243 |
| | 587 | - | 1.40 | 6.00 | 18.00 | 1.058 |
| | 627 | - | 1.41 | 6.00 | 18.00 | 1.085 |
| DMF | 547 | - | 1.43 | 6.00 | 19.00 | 1.403 |
| | 587 | - | 1.05 | 6.00 | 19.00 | 1.297 |
| | 627 | - | 1.00 | 6.00 | 19.00 | 1.156 |
| Hexane | 547 | 0.51 | 1.84 | 6.10 | - | 1.222 |
| | 587 | - | 1.81 | 6.25 | - | 1.300 |
| | 627 | - | 1.94 | 6.33 | - | 1.277 |
| Cyclohexane | 547 | 0.54 | 2.05 | 6.00 | - | 1.324 |
| | 587 | - | 2.15 | 6.00 | - | 1.235 |
| | 627 | - | 2.27 | 6.00 | - | 1.202 |
| Benzene | 547 | - | 2.90 | 6.00 | 21.00 | 1.212 |
| | 587 | - | 3.04 | 6.00 | 21.00 | 1.207 |
| | 627 | - | 3.19 | 6.00 | 21.00 | 1.209 |
| Toluene | 547 | 0.16 | 3.74 | 6.00 | 21.20 | 1.145 |
| | 587 | - | 3.70 | 6.00 | 21.20 | 1.176 |
| | 627 | - | 4.13 | 6.00 | 21.20 | 1.122 |
| Xylene | 547 | 0.05 | 3.77 | 6.00 | 21.50 | 1.245 |
| | 587 | 0.06 | 4.03 | 6.00 | 21.50 | 1.146 |
| | 627 | - | 4.27 | 6.00 | 21.50 | 1.167 |

**Table S5.** Values of time constants ($\tau_i$) of the emission decay of 025P@MIL-68(In) powder and its pellets upon excitation at 445 nm.

| **Sample** | **Wavelength (nm)** | **$\tau_1$ (ns)** | **$\tau_2$ (ns)** | **$\tau_3$ (ns)** | **$\tau_4$ (ns)** | $\chi^2$ |
|---|---|---|---|---|---|---|
| Powder | 547 | 0.56 | 3.00 | 7.00 | 18.20 | 1.276 |
| | 587 | 0.66 | 3.00 | 7.00 | 18.20 | 1.200 |
| | 627 | 0.53 | 2.70 | 6.65 | 18.20 | 1.332 |
| 346.60 MPa | 547 | 0.39 | 2.53 | 6.86 | 19.50 | 1.269 |
| | 587 | 0.54 | 2.83 | 6.91 | 19.50 | 1.280 |
| | 627 | 0.50 | 2.85 | 6.89 | 19.50 | 1.144 |
| 693.20 MPa | 547 | 0.38 | 2.48 | 6.85 | 20.00 | 1.253 |
| | 587 | 0.57 | 2.96 | 6.92 | 20.00 | 1.291 |
| | 627 | 0.38 | 2.73 | 6.83 | 20.00 | 1.260 |

**References:**

[1] Fajal, S.; Mandal, W.; Majumder, D.; Shirolkar, M. M.; More, Y. D.; Ghosh, S. K., Unfolding the Role of Building Units of MOFs with Mechanistic Insight Towards Selective Metal Ions Detection in Water. *Chem. Eur. J.* **2022,** 28, 202104175.
